# Needs-led research: a way of employing user involvement when devising research questions on the trust model in community home-based health care services in Norway

**DOI:** 10.1186/s40900-021-00291-0

**Published:** 2021-06-22

**Authors:** Ruth-Ellen Slåtsveen, Torunn Wibe, Liv Halvorsrud, Anne Lund

**Affiliations:** 1 Department of Occupational Therapy, Prosthetics and Orthotics, Faculty of Health Sciences, Oslo Metropolitan University, PO Box 4, St. Olavs Plass, 0130 Oslo, Norway; 2 Centre for Development of Institutional and Home Care Services in the City of Oslo, PO Box 435, Sentrum, 0103 Oslo, Norway; 3 Department of Nursing and Health Promotion, Faculty of Health Sciences, Oslo Metropolitan University, PO Box 4, St. Olavs Plass, 0130 Oslo, Norway

**Keywords:** Needs-led research, User involvement, James Lind Alliance, Home-based health care services, Trust model

## Abstract

**Background:**

This paper presents a user involvement process, called needs-led research, conducted as a part of a doctoral degree project aiming to explore research priorities and, ultimately, to develop a final top 10 list of questions relevant to the field of research. There is evidence of a mismatch between what user groups within a research field find relevant to study and what is actually being done. User involvement is a method that can accommodate this, and there is a growing attention and amount of research in this field based on an understanding that people who receive health care services, and their next of kin and clinicians, are uniquely positioned to contribute to research in order to understand their experiences better and improve the services. This paper presents a user involvement process in a small-scale study, referred to as needs-led research, which concerns the ‘performance of the trust model in community home-based health care services’. The process was conducted as part of a doctoral degree project.

**Method:**

The needs-led research process is inspired by the James Lind Alliance (JLA), which focuses on bringing together service users, next of kin and clinicians on equal terms to explore research priorities. The process consisted of five-steps, each of which involved representatives from service users, next of kin and clinicians: 1) narrowing down the theme; 2) steering group meeting; 3) gathering input through a survey; 4) data processing and interim priority setting; and 5) final priority setting.

**Results:**

Almost 200 participants contributed during the five steps, 294 inputs were gathered, and 35 participants voted for the top 10 list. The top 10 list is presented.

**Conclusion:**

This paper provides an example of how user involvement can be employed to devise research questions that are relevant for clinicians, service users, next of kin and service providers concerning the ‘performance of the trust model in home-based health care’. It also outlines some strengths and limitations of the process. The needs-led research process shows that user involvement in research is feasible for developing research questions in small-scale studies. We hope that the top 10 list presented will encourage future research to address issues of importance regarding the performance of the trust model in community home-based health care services.

## Plain English summary

User involvement is a topic that has received increasing attention in research aimed at ensuring relevant and needed knowledge. It can be practised in a variety of ways, and the literature claims that there is no gold standard for how it should be done. This paper presents a user involvement process conducted autumn 2019 to spring 2020, hereby referred to as needs-led research (NLR), inspired by the James Lind Alliance (JLA). The scope of the process is the ‘performance of the trust model in community home-based health care services’. The trust model is a new way of organising home-based health care services where small interdisciplinary teams work with the service users and their next of kin on tailoring the services based on the users’ answers to the question ‘What matters to you?’

Through a five-step process, in which representatives of service users, next of kin and clinicians participated in meetings and online surveys, the goal of the NLR process was to explore research priorities and, ultimately, to develop a final top 10 list of questions relevant to the field of research. This paper describes the NLR process and discusses its strengths and limitations. We found the process to be important when identifying relevant research questions concerning a topic on which little research has been conducted, and in which we as researchers have little experience. The final top 10 list is also presented.

## Background

This paper presents a user involvement process, called needs-led research (NLR), conducted as a part of a doctoral degree project, from autumn 2019 to spring 2020, aiming to explore research priorities and, ultimately, to develop a final top 10 list of questions relevant to the field of research. There is evidence of a mismatch between what user groups in a research field find relevant to study and what is actually being done [1–3]. User involvement has been proposed as a method to accommodate this, and there is a growing attention and amount of research in this field, based on an understanding that the people who receive health care services, and their next of kin and clinicians, are uniquely positioned to contribute to research aimed at improving the health care services [4–9]. There are many concepts and definitions related to user involvement, but in this article, we define it as ‘research being carried out “with” or “by” members of the public or users rather than “to”, “about” or “for” them’ [10]. Equitable involvement of service users and clinicians when setting research priorities is ethically desirable and is said to improve the quality, relevance and implementation of research [5, 11]. A key argument for involving users in the research field is to ensure participatory democracy, which is supported by ethical arguments and enshrined in human rights [12]. It is also reported to generate better and more relevant decisions, and to create better opportunities and support for further work or changes if needed [12]. There are probably as many approaches to user involvement in research prioritisation as there are priority-setting exercises, and a gold standard is therefore not attainable, nor is it appropriate [13]. The NLR process described in this paper was inspired by the James Lind Alliance (JLA) which focuses on bringing together service users, next of kin and clinicians on equal terms to explore research priorities in a priority-setting partnership by asking what matters to them the most [1]. JLA is designed to lead to changes in the way research funding is granted and is a thorough and time-consuming model that can last up to 18 months [1]. Since this was a part of a doctoral degree project, it was necessary to make some pragmatic choices and adjustments to make the model feasible in a small-scale study. Thus, JLA was used as an inspiration when it came to establishing a collaboration with representatives of service users, next of kin and clinicians, focusing on what matters to them, and prioritising a top 10 list of important areas for research [1]. Although user involvement is applied in many studies, detailed descriptions of the involvement process and a robust evidence base for its impact are still lacking [8, 14, 15]. In this paper, we describe a process of user involvement intended to explore the research priorities of people with experience of the trust model in community home-based health care services, including representatives from the health care service users, their next of kin, and clinicians from these services in a relatively large municipality in Norway. The trust model is a new way of organising home-based health care services where trust is described as a strategy and work method aimed at involving service users and next of kin in decisions that concern them, and trusting clinicians’ professional judgement to a greater extent when it comes to assessing the need for services and adjusting them if the user’s health changes. This thereby makes the services individually tailored and more flexible [16]. Decisions are made in small, interdisciplinary teams, that include case officers, and these also perform the services. The trust model is intended to create flexibility with the possibility of following up on changing needs for the individual service user based on professional discretion and what the users perceive as important to them [16]. The scope of the study was the ‘performance of the trust model in community home-based health care services’, an area that has received limited attention in research, both with respect to Norway and internationally. No systematic reviews are available and only a few small-scale studies have been conducted. The trust model constitutes a new way of organising home-based health care services, and little knowledge exists about what the service providers employing the model, or the service users and next of kin, consider relevant to study, making it appropriate to implement a user involvement process.

### Aim

The aim of this paper is to describe a way of applying NLR in a small-scale study and to address strengths and limitations of this way of employing user involvement. A top 10 list of research questions generated through the NLR process will also be presented.

## Method

**Fig. 1:**
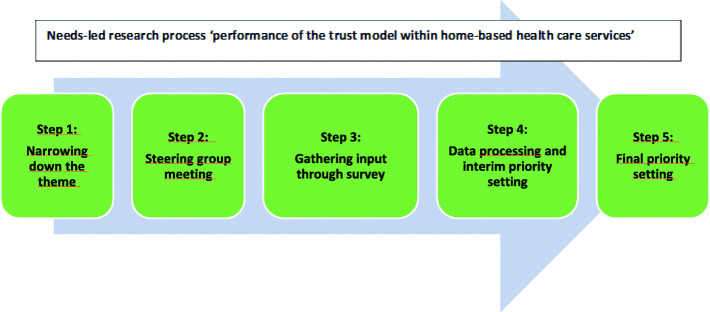
NLR process flowchart

The needs-led research (NLR) process is inspired by James Lind Alliance (JLA). The characteristics of the JLA process are: 1) creating a steering group; 2) gathering evidence uncertainties; 3) summarising the responses gathered; 4) evidence checking; 5) interim priority setting; 6) workshop to conduct the top 10 list; and 7) publishing and promoting the top 10 list of research priorities [1]. This project’s NLR process is outlined in Fig. 1 and consisted of five steps; 1) narrowing down the theme; 2) steering group meeting; 3) gathering input through a survey; 4) data processing and interim priority setting; and 5) final priority setting.

The difference between these two processes is the number of users involved, the time spent, the way of summarising/processing the data and interim priority setting, and how the top 10 list was compiled. JLA uses the term ‘evidence uncertainties’ to define the unanswered questions that are identified and prioritised during this process [1]. These are referred to as ‘input’ in this paper.

The scope was the ‘performance of the trust model in community home-based health care services’, with representatives of service users, next of kin and clinicians as participants in the NLR process. Throughout the five steps, efforts were made to involve an appropriate and balanced representation of the representatives in order to minimise the risk of overlooking research options and to make sure that the prioritisation corresponded to the needs of those who will benefit from the research priorities [13].

### Step 1: narrowing down the theme

For the purpose of narrowing down the theme, clinicians, organisations for service users and next of kin, members of senior citizens’ councils and the Patient Ombudsman, all with connections to the municipality chosen for the project, were invited to a workshop. The aim was to generate ideas on important aspects of the overall theme [13]. We wanted the representatives to suggest areas they considered most relevant for research [15]. Eighteen representatives participated, who were evenly distributed among the groups they represented. The first author started the workshop with a presentation of the meeting agenda and some general information including the aim of the workshop. The participants were then divided into three groups. Each group consisted of representatives of service users, next of kin and clinicians, and had a group leader and a minute taker.

Firstly, the participants were invited to write down key words on themes, thoughts and experiences regarding the scope. Yellow post-it notes were used for this exercise, which lasted about 5 min. Secondly, the participants shared and elaborated their notes with the others, and simultaneously organised the key words they thought belonged together or had similar systems on a larger white piece of paper they were given beforehand. Thirdly, the participants discussed the notes and were asked to reformulate their groupings into areas or themes using pink post-it notes. The session and discussion in each group lasted for approximately 1 h.

The aim of this session was to create an opportunity for everyone to talk and elaborate on important aspects of employing the trust model. Inspired by qualitative research, the goal was to promote dialogue between the participants [17]. They listened to each other’s stories, and offered comments and sometimes different perspectives, which led to rich descriptions and potentially new understandings [17]. To ensure a safe environment in which all the participants could talk, we started out with small groups. According to Korstjens and Moser [18], the advantage of small groups is that they give participants more time to elaborate on their perspectives and stories, and they may therefore contribute more detailed information. The feedback received afterwards the session was that the group composition and dynamics were good, and many felt that they benefited from sharing experiences and thoughts with each other in this way. The representatives took the opportunity to ask each other questions about the different groups of service users, issues regarding home-based services, and especially the involvement of service users and next of kin within the services. All three groups reported active participation from all of the representatives. After a break, everyone came together and each group presented its topics, thoughts and/or experiences. Many of the areas were similar, and there were no major differences in the topics the groups had discussed. A discussion was then initiated in the larger group, and several participants continued to share and problematise the performance of the trust model. This helped to create a form of consensus about the areas and themes that had been raised [18]. The aim of the meeting was to reach consensus about one or more themes to proceed with in the needs-led research process, but this was not possible. we obtained 73 inputs, which provided direction for further focus. In addition, important and useful input related to the performance of the trust model was received, and several connections to the field were established.

### Step 2: steering group meeting

The term steering group is inspired by JLA [1] with the aim of ensuring user involvement in all steps of the process. The group members were not co-researchers, but rather a group that could provide consultation, input and a connection to the field, as well as influence the steps of the process and the choices made. As experts in their field, they were asked to contribute their insight and knowledge about the group within the research field they represented. They were familiar with current debates through their affiliation with patient organisations [12] or the home-based services. The steering group consisted of six members: three representatives from the home-based health care services, two service user representatives and one next of kin. Input from the brainstorming session was presented and discussed and served as the basis for the next step. There was consensus among the steering group members that the focus should be on the clinician/user level. The steering group members were concerned about whether the trust model provided more accurate and better services, more service user and next of kin involvement, and how the work was performed in practice. This was the starting point for the online survey questions in Step 3. The questions were formulated and accepted by the members present at the steering group meeting. A summary and a draft of the survey were subsequently distributed to all the members, offering them the opportunity to comment on the topics being discussed or on the questions in the survey. We received comments from one member, representing the home-based health care services, related to wording in the survey, and the text was altered accordingly.

### Step 3: gathering input through an online survey

The survey was kept short, with brief, to-the-point information about the study and a few checkboxes asking for the type of representative, gender, profession (if clinicians) and degree of familiarity with and experience of the trust model. This was followed by brief information about what the services should contain and what they should deliver within the trust model. The information was based on the overall standards for community home-based health care services the municipality in the project itself had published. There were two versions of the main question: one for clinicians and one for service users and next of kin.

The clinicians were asked: ‘*Home-based health care services should be provided through small teams with more user involvement, based on the question “What matters to you?”, with more competent and empowered staff, and with increased interdisciplinary cooperation. If you think about your experience with home-based health care services, what do you think we should investigate in relation to this area?’*

The service users and next of kin were asked: *‘Home-based health care services should be provided through small teams where the user and relatives should help determine what the service should be, based on the question “What matters to you?”. You should have competent service providers who can make decisions with you when it comes to your services or the services of the person to whom you are next of kin. The staff will work across professions to provide you with good services that are interconnected. If you think about your experience with home-based health care services, what do you think we should investigate in relation to this area?’*

The trust model was deliberately not mentioned in the question, based on feedback from the steering group. Most of the service users and next of kin are unlikely to have heard about the trust model and would therefore lack sufficient knowledge to comment on it. The representatives from the services agreed, since several of the clinicians were also likely to have difficulties answering what the trust model comprised. Therefore, the decision was made to focus on what the services should contain or deliver. The questions were open-ended to encourage full responses regarding the experiences and perceptions of service users, next of kin and clinicians [18].

When promoting the online survey, the organisations that participated in the workshop (Step 1) shared information and links to the survey with their members using their existing communication platforms. Centrally located clinicians in the municipality received information and the link via e-mail and distributed them in appropriate networks within the community. This ensured that all leaders in home-based health care services received the information and were encouraged to both respond to and share the link with their employees.

**Fig. 2:**
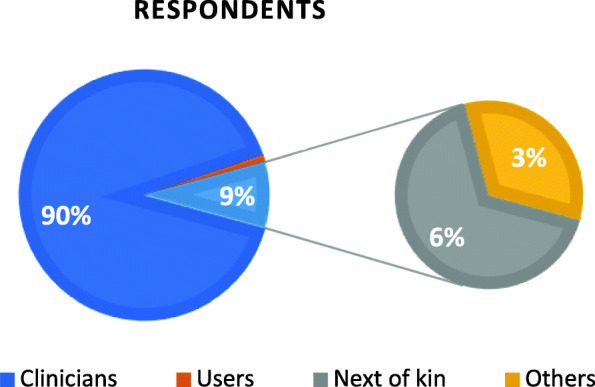
Distribution of respondents

The questionnaire was published in early January and stayed open for 4 weeks, and 106 responses comprising 221 inputs were collected. Most of the respondents were representatives of the home-based health care services (Fig. 2).

Respondents representing clinicians were leaders, occupational therapists, physiotherapists, nurses, nutritionists and unskilled workers.

### Step 4: data processing and interim priority setting

The aim of this step was to review the inputs – 73 from Step 1 and 221 from Step 3 – and organise them into thematic groups. Many of the inputs were formulated as comments or statements rather than questions. To accommodate and include these, a thematic approach was found appropriate. The researchers went through the inputs, grouping and discussing them, and seven themes were created along with 16 research questions during this process (see Table 1 for an overview of the themes with examples of input). The steering group was involved in the process of verifying the interpretations and clarifying the questions. They received all the input, groupings and questions that were created and were asked to provide feedback. One piece of feedback was received concerning ease of understanding in a few of the questions, which were altered accordingly.

**Table 1:** Examples from the thematisation process

Themes	Examples of input	Examples of research questions
Interdisciplinary collaboration	- What are success criteria for successful interdisciplinary cooperation? - How is systematic work in interdisciplinary collaboration facilitated organisationally? - The importance of interdisciplinarity in the teams?	How is interdisciplinary collaboration performed in small teams?
Competence/ Knowledge	- Has it become easier or more difficult to ensure that personnel with the right competence are assigned to patients where this competence is necessary? - Core competence: To clarify what competence the service needs for different positions and roles in order to have competent and empowered employees: e.g. observational competence of employees - Competence to assess the user’s functional ability and cognitive status?	What interdisciplinary competencies do the employees in the teams need, and how do they make best possible use of these competencies?
Assistive technology and digitalisation in the service	- More on assistive technology because more things are becoming electronic and there are still many who have not understood that it will become more digital with time. - Training and information about it should be more prioritised. - Research more on how different departments work on the services	How are assistive technology and digitalisation implemented and used within interdisciplinary teams?
Organisations and leadership	- How does the trust model contribute to an organisation that provides more tailored and flexible services? - What significance do organisation and management have for the performance of small interdisciplinary teams?	
Early intervention/ preventive services	- Is there still more focus on nursing than on rehabilitation? - Do they manage to integrate reablement as well? - The rehabilitation services in specialist health care are reduced, and it is expected that the municipalities will provide rehabilitation to a greater extent. The districts are falling behind in stepping up rehabilitation services to meet these requirements (of the trust model). How will the districts manage to step up and organise the service to meet users in need of rehabilitation?	How does the trust model ensure early intervention and preventive work?
Exercise of authority and profession	- Roles and responsibilities in the trust model – how has it affected employees and the organisation? - The term ‘empowered employees’ and independent professional practice is often used in connection with the trust model. Do employees feel that they have been given more authority and a greater degree of independence in their professional practice? Do the employees experience wider scope of action?	How are roles and responsibilities exercised by employees in the trust model?
User and next of kin involvement	- The goal of having small teams and a few people to relate to is not implemented in practice. This causes frustration among both users and next of kin. - How the services involve relatives in planning, design, implementation and evaluation of the services and how next of kin experience it? - Whether the trust model gives users an experience of better interdisciplinary collaboration and fewer people to relate to, whether they are assigned a ‘primary contact’ and whether they have good contact with her/him.	How do users and next of kin experience involvement in the process of allocating services when exercising the trust model?

### Step 5: final priority setting

The aim of the final priority setting was to develop a list of the top 10 research questions on the ‘performance of the trust model in community home-based health care services’. The object of the list is to highlight important areas for research, while not necessarily devising the specific research questions [1]. Invitations were widely distributed to service users, next of kin and clinicians in March, but because of the Covid-19 outbreak, the planned workshop for final priority setting had to be cancelled. Zoom and Teams were not considered to this end due to the fact that these platforms were not accessible at that time. An online survey was therefore conducted based on the 16 questions from the interim priority setting. The respondents were asked to select a maximum of 10 questions they found the most important and to comment on why they considered them important. The survey was widely distributed to organisations for service users and next of kin, members of senior citizens’ councils, the Patient Ombudsman and clinicians. The survey was open for 14 days, and 35 responses were received representing employees, users, next of kin and others.

**Fig. 3:**
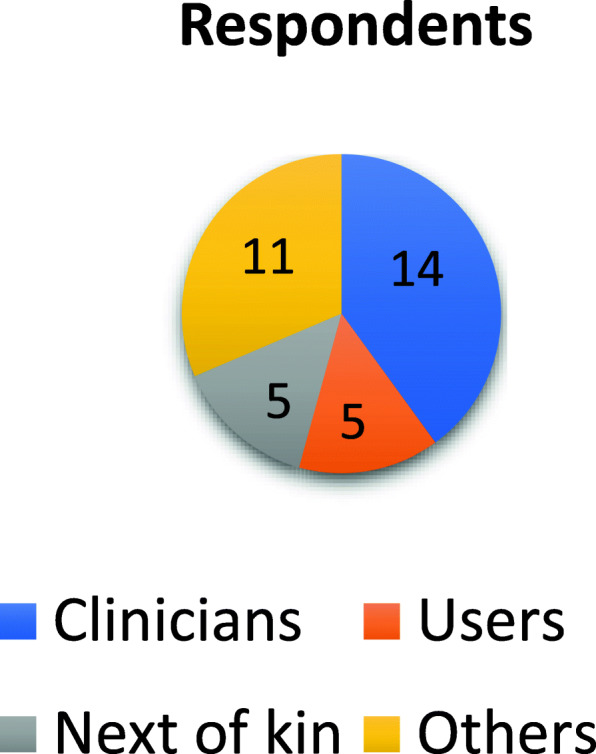
Distribution of respondents in final priority setting

‘Others’ represents e.g. members of senior citizens’ councils, chairs of supervisory committees for short-stay institutions and nursing homes, politicians and volunteers. Before the top 10 list was published, it was submitted to the steering group in order to verify the list’s relevance by the representatives (Fig. 3).

### Results: the top 10

The votes for the top 10 were evenly distributed, all rating above 40%, and the question voted in second place was selected as the main question for this doctoral degree project:

*How does the trust model contribute to flexible and individually tailored services?*

**Fig. 4:**
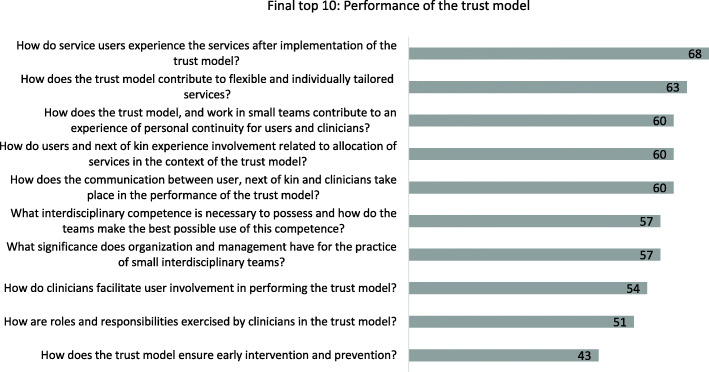
The top 10 research question regarding performance of the trust model, % voting

We hope that this list (Fig. 4)  will encourage to future research that addresses issues of importance regarding the performance of the trust model in community home-based health care services.

## Discussion

The aim of this paper was to describe a way employing NLR in a small-scale study. User involvement is seen to represent a cultural change in health care, and requires commitment, time, sensitivity and a willingness to experiment and learn from mistakes [19]. In order to address some of these challenges, this section will discuss some of the strengths and limitations of the process in order to provide relevant insights for others who might choose a similar method, and to address the identified knowledge gap concerning challenges and recommendations related to user involvement processes [20].

We have strived to achieve an appropriate representation of service users, next of kin and clinicians during the NLR process to minimise the risk of research questions being overlooked, to foster interest and a desire to be a part of the ongoing doctoral degree project, and to align with the needs of those who will implement and benefit from the research priorities [13]. There has been broad participation from the health care services. Although great efforts were made to include service users with experience and knowledge of the trust model, the number of service user respondents was low, especially when obtaining input through the online survey in Step 3. Invitations were distributed online to, and promoted on social media by, interest organisations such as pensioner associations and the Norwegian Public Health Association.

In a relatively small-scale project such as the present study, where time and resources are limited, it was not feasible to reach more service users or to run a larger campaign to attract them. Those who participated as service users were representatives, such as senior citizens, members of senior citizens’ councils, the ombudsman for senior citizens and members of relevant local government committees for senior citizens. These might not represent the wider user population receiving home-based health care services. However, by comparing their input with that received from next of kin, we identified overlapping areas of interest. This may indicate that the user representatives have insight into the services, challenges and knowledge needs of the service users themselves. It is highly likely that we did not reach the silent voices when conducting this process, i.e. individuals in low socioeconomic groups, with low health literacy or who have no access to the internet and social media [21]. Culture and language are other barriers that need to be addressed [22], in terms of how to create good information in other relevant languages and where to distribute this information in order to reach a larger group of people. Consideration should be given to identifying input from the service users separately if conducting a similar process in a larger project and involving other relevant representatives from different cultures and languages.

Critics have argued that, despite its democratic intentions, this way of employing user involvement does not necessarily empower patients, since the researcher retains – and may choose to wield – her power to define what a legitimate research question is and how to answer it [23]. Attempts have been made to eliminate this by involving the steering group after the interim priority setting, and when the final research questions were devised. Nonetheless, we cannot claim that the silent voices have been reached and heard. Another aspect to point out is that the greatest influence during the last steps of the process was held by the authors. To avoid this bias, the steering group members could have actively participated in the process of thematising, interpreting and developing the questions in the interim priority setting during Step 4. Inviting some of the steering group members or another representative to be a co-author of this article could also have contributed to richer and more nuanced perspectives and experiences of this NLR process. This would raise issues such as increased time consumption, the question of financially compensating the members, and finding members with sufficient time and interest to participate in such processes. Time and cost are enlightened in other studies as important aspects of user involvement in research [24]. The users and next of kin representatives in the steering group were offered reimbursement for travel to attend the meeting in Step 2, but no other honorariums were offered. Those representing the services attended the meeting during their working hour. In projects with a higher budget and thereby possibilities to provide honorariums to those who use their spare time for this kind of work, honorarium must be considered. This is not only for acknowledging the time, resources and expertise given to the project but also to create a sense of equality among the members.

An evaluation could have been carried out of the process and of the representatives’ involvement to increase the quality and acceptability, and this may well have provided insight into the experience of participating in such a process and its perceived impacts [13, 25]. In order to avoid tokenism and a tick-box approach [23, 26], the participants and the feedback provided during this process have been given a strong influence. Since the researchers have no experience of the trust model or its organisational form, the NLR process ensured that the top 10 list was relevant, and that it was grounded in the day-to-day reality of service users, their next of kin and clinicians [27, 28]. JLA recommends removing non-questions, such as statements and comments [1], but since many of the inputs received through the questionnaire in Step 3 were not formulated as questions, we decided to include them. One strength of this process is that every input was grouped, while a limitation lay in the interpretation that was needed when grouping the input and creating questions that captured their essence. Attempts were made to keep the questions as close to the input as possible, but some adaptations were necessary (see Table 1). All inputs were assigned equal importance, regardless of which groups they represented. Input from clinicians was often related to the trust model and its organisation and performance, while input from the service users and next of kin was related to their experiences, as well as seeking more knowledge about performance. Although no distinctions were drawn between the user groups when interpreting the input, we found similar interests between them. Consensus was also found in the prioritisation of the top 10 list. Votes for the research question that was finally selected – ‘*How does the trust model contribute to flexible and individually tailored services?’ –* received seven votes from service providers, four from next of kin, one from a service user and eight from representatives of users and next of kin (e.g. members of senior citizens’ councils, politicians and interest organisations). This distribution of votes underpins the research relevance for the field and supports Brett et al.’s finding of the positive impact of user involvement on identifying user-relevant topics for research agendas and priorities [24].

Although the outbreak of the Covid-19 pandemic created certain limitations, it may also have created some strengths. JLA recommends that the final priority setting be conducted as a workshop with balanced participation of service users, next of kin and service providers [1], where optional group techniques are presented in order to promote good reflections and discussions. This was not feasible because of the national lockdown during the Covid-19 pandemic, and an online survey was conducted instead. Despite the lack of opportunities for discussion and knowledge sharing within the group [29], the online survey attracted more participants, and several of them gave reasons for their chosen prioritisation. At the same time, bias was avoided by preventing the first speaker from setting the agenda and influencing the opinions of the other participants. The participants could vote freely based on their own opinions, and no one was able to know how they voted. The method of prioritisation may have contributed to providing a safer context for the participants. Fifteen participants had signed up for the planned workshop, whereas 35 participants completed the online survey.

The top 10 list reflects a need to address clinicians’ competence and knowledge, their approach to and involvement of service users and next of kin, and the organisational structure of the trust model in research.

In addition to identifying research questions that are relevant to the field of research, user involvement is also seen as filling gaps in researchers’ knowledge and correcting assumptions, thereby avoiding bias in researchers’ ways of thinking [15]. This NLR process generated new knowledge for us as researchers, not only regarding the trust model and its organisational framework, but also regarding the different perspectives and experiences held by service users, next of kin and clinicians. Without user involvement, the research questions devised are likely to have been different. This NLR process is not tested or validated any further than this project and paper. We strongly advise further reporting on the process if implemented in other projects in order to gain a larger insight and knowledge of needs-led-research.

## Conclusions

In a time when user involvement should be a goal in every study, this paper provides an example of how to employ user involvement in order to devise research questions that are relevant for clinicians, service users and next of kin, and for the service providers themselves regarding the ‘performance of the trust model in community home-based health care services’. The paper presents a needs-led research process conducted as part of a doctoral degree project and shows that user involvement in research is feasible for developing research questions in small-scale studies. It also outlines strengths and limitations of the process and present the top 10 list of relevant research question. It is our hope that the list will encourage future research to address issues of importance regarding the performance of the trust model, which is a relatively new way of organising community home-based health care services in Norway.

## Data Availability

The material in this project is material from workshops and surveys, all in Norwegian.

## Abbreviations

NLR: Needs-led research
JLA: James Lind Alliance

## References

[1] National Institute for Healt Research (2020). The James Lind Alliance guidebook version 9.

[2] Glasziou P, Chalmers I (2018). Research waste is still a scandal—an essay by Paul Glasziou and Iain Chalmers. BMJ.

[3] Crowe S, Fenton M, Hall M, Cowan K, Chalmers I (2015). Patients’, clinicians’ and the research communities’ priorities for treatment research: there is an important mismatch. Res Involv Engagem.

[4] Hanley B, Truesdale A, King A, Elbourne D, Chalmers I (2001). Involving consumers in designing, conducting, and interpreting randomised controlled trials: questionnaire survey. BMJ.

[5] Evans I (2011). Testing treatments: better research for better healthcare.

[6] Shippee ND, Domecq Garces JP, Prutsky Lopez GJ, Wang Z, Elraiyah TA, Nabhan M, Brito JP, Boehmer K, Hasan R, Firwana B, Erwin PJ, Montori VM, Murad MH (2015). Patient and service user engagement in research: a systematic review and synthesized framework. Health Expect.

[7] Harrison JD, Auerbach AD, Anderson W, Fagan M, Carnie M, Hanson C, Banta J, Symczak G, Robinson E, Schnipper J, Wong C, Weiss R (2019). Patient stakeholder engagement in research: a narrative review to describe foundational principles and best practice activities. Health Expect.

[8] Dawson S, Ruddock A, Parmar V, Morris R, Cheraghi-Sohi S, Giles S (2020). Patient and public involvement in doctoral research: reflections and experiences of the PPI contributors and researcher. Res Involv Engagem.

[9] Thornton H, Edwards A, Elwyn G (2003). Evolving the multiple roles of ‘patients’ in health-care research: reflections after involvement in a trial of shared decision-making. Health Expect.

[10] National Institute for Health Research. What is public involvement in research? NHIR/INVOLVE2020. Available from: https://www.invo.org.uk/find-out-more/%20what-is-public-involvement-in-research-2/. Accessed 09 July 2020.

[11] Alex P, Bridget St G, Mark F, Sally C, Lester F (2014). Development of a new model to engage patients and clinicians in setting research priorities. J Health Serv Res Policy.

[12] Uhm S, Liabo K, Stewart R, Rees R, Oliver S. Patient and public perspectives shaping scientific and medical research: panels for data, discussions, and decisions. Patient Intell. 2012;4:1–10.

[13] Viergever R, Olifson S, Ghaffar A, Terry R (2010). A checklist for health research priority setting: nine common themes of good practice. Health Res Policy Syst.

[14] Smits D-W, van Meeteren K, Klem M, Alsem M, Ketelaar M (2020). Designing a tool to support patient and public involvement in research projects: the Involvement Matrix. Res Involv Engagem.

[15] Staley K, Barron D (2019). Learning as an outcome of involvement in research: what are the implications for practice, reporting and evaluation?. Res Involv Engagem.

[16] Eide T, Gullslett MK, Nilsen E, Dugstad JH, Eide H. Tillitsmodellen: hovedpilotering i Oslo kommune 2017 –18 [The trust model: main pilot in Oslo municipality]: Universitetet i Sørøst-Norge. Vitensenteret helse og teknologi;2018. Available from: http://hdl.handle.net/11250/2583361.

[17] Tjora AH (2019). Qualitative research as stepwise-deductive induction.

[18] Korstjens I, Moser A (2017). Series: practical guidance to qualitative research. Part 2: context, research questions and designs. Eur J Gen Pract.

[19] Pepper L (2018). Patient and public involvement in sexual and reproductive health: a new editor, and a new tool. BMJ Sex Reprod Health.

[20] Pii KH, Schou LH, Piil K, Jarden M (2019). Current trends in patient and public involvement in cancer research: a systematic review. Health Expect.

[21] Nygaard A, Halvorsrud L, Linnerud S, Grov EK, Bergland A (2019). The James Lind Alliance process approach: scoping review. BMJ Open.

[22] Staniszewska S, Brett J, Simera I, Seers K, Mockford C, Goodlad S (2017). GRIPP2 reporting checklists: tools to improve reporting of patient and public involvement in research. BMJ.

[23] Greenhalgh T, Hinton L, Finlay T, Macfarlane A, Fahy N, Clyde B, Chant A (2019). Frameworks for supporting patient and public involvement in research: systematic review and co-design pilot. Health Expect.

[24] Brett J, Staniszewska S, Mockford C, Herron-Marx S, Hughes J, Tysall C (2014). A systematic review of the impact of patient and public involvement on service users, researchers and communities. Patient.

[25] Nunn JS, Shafee T, Chang S, Stephens R, Elliott J, Oliver S, et al. Standardised Data on Initiatives - STARDIT: Alpha Version (Preprint) 2019. (Cited 2020 Oct 07). Available from: 10.31219/osf.io/5q47h.

[26] Malterud K, Elvbakken KT. Patients participating as co-researchers in health research: a systematic review of outcomes and experiences. Scand J Public Health. 2019:140349481986351. 10.1177/1403494819863514. 10.1177/1403494819863514 31319762

[27] Snow R, Crocker JC, Crowe S (2015). Missed opportunities for impact in patient and carer involvement: a mixed methods case study of research priority setting. Res Involv Engagem.

[28] Staley K (2015). ‘Is it worth doing?’ Measuring the impact of patient and public involvement in research. Res Involv Engagem.

[29] James Lind Alliance (2021). JLA Lab activity 1: development of online priority setting workshop. Lessons learned. Report February 2021.

